# The effect of internet use on depressive symptoms in middle-aged and older adults with functional disability: the mediating role of social isolation

**DOI:** 10.3389/fpubh.2023.1202541

**Published:** 2023-07-10

**Authors:** Man Wu, Chaoyang Li, Xueyang Zhao, Ting Hu, Lijuan Zeng, Yiqing Yu, Fen Yang, Yangyang Han

**Affiliations:** ^1^School of Nursing, Hubei University of Chinese Medicine, Wuhan, China; ^2^School of Nursing, Shandong First Medical University and Shandong Academy of Medical Sciences, Taian, China

**Keywords:** internet use, depressive symptoms, functional disability, social isolation, middle-aged and older adults

## Abstract

**Objective:**

The popularization of the internet provides the possibility to improve the depressive symptoms (DS) and social isolation of middle-aged and older adults with functional disability. There was a significant correlation between internet use and DS in middle-aged and older adults with functional disability, but the relationship between internet use, social isolation, and DS remains to be confirmed.

**Methods:**

Data were obtained from 7,955 middle-aged and older adults aged 45 years and older from the 2018 China Health and Retirement Longitudinal Study (CHARLS). Multiple linear regression models were used to analyze the association between internet use and DS, and the Sobel test was used to explore mediating models.

**Results:**

Results showed that internet use was negatively associated with DS among Chinese middle-aged and older adults. Among them, social needs (B = −0.770, *p* = 0.041), and information reception (B = −1.067, *p* = 0.007) were significantly related to DS in middle-aged and older adults. Only information reception (B = −3.161, *p* = 0.031) was significantly related to DS among middle-aged and older adults with functional disability. Social isolation partially mediated the association between internet use and DS in middle-aged and older adults without functional disability, whereas it was fully mediated in middle-aged and older adults with functional disability.

**Conclusion:**

This study indicates that when formulating health policies to promote the mental health of Chinese middle-aged and older adults, the use of the internet should not be overlooked.

## 1. Introduction

Depressive symptoms (DS), as a common psychiatric problem, are projected to become the major burden of disease worldwide by 2030 (1). DS are associated with increased mortality, functional disability, and a poor prognosis for many other diseases (2). The manifestation of DS in middle-aged and older adults has garnered extensive societal attention. Worldwide, the prevalence of DS reaches its peak in individuals aged 55–74 years (1). In China, the combined prevalence of DS among middle-aged and older adults is significantly high, reaching up to 22% (3), with approximately 30% of men and 43% of women in the 45 and older age group experiencing such symptoms (4). The DS of middle-aged and older adults are usually a comprehensive result of physical, psychological, and social factors related to aging (5). Many late-life DS seem to be attributable to functional limitations caused by physical illness (6). Functional disability is considered to be the most important predictor of DS in middle-aged and older adults (7). There is growing evidence that activities of daily living/instrumental activities of daily living (ADL/IADL) disability is associated with DS (8). The prevalence of DS is higher among middle-aged and older Chinese with functional disability compared to those without such disability (8, 9). For each additional ADL/IADL disability type, the risk of DS in middle-aged and older adults increases by more than 2.6 times (7). People with functional disability may amplify DS by reducing perceived social support and psychological resources (10). Persistence of DS may lead to progressive deterioration of functional status (11). For middle-aged and older adults with functional disability, DS have long-term negative effects on their occupational roles, social adjustment, leisure activities, and family relationships (7). Not only does it reduce their psychosocial functioning and quality of life (12, 13), it can even lead to an increased risk of suicide (14). Therefore, it is critical to identify potentially modifiable factors of DS in middle-aged and older adults with functional disability. With the development of network information, the internet’s functional versatility, immediacy, and accessibility offer the possibility to alleviate DS in middle-aged and older adults with functional disability.

Internet use is both a challenge and an opportunity for middle-aged and older adults with functional disability. With the increase of internet penetration rate, the scale of internet use among middle-aged and older adults is continuing to expand. There is growing concern about the potential adverse effects of age-related changes in physical functioning and the digital divide on internet use among middle-aged and older adults (15). Studies show that adults with ADL/IADL disability are significantly less likely to be online than other adults (54% vs. 81%) (16). Compared to other age groups, internet use rate is much lower in middle-aged and older groups where functional disability is most common (17). Due to the vulnerability of such groups in accessing and using communication, it may further exacerbate the inequality of information (18). Nevertheless, internet use has also brought benefits to such groups. The internet, as a comprehensive platform that integrates information, social interaction, and entertainment, can better meet the diverse needs of middle-aged and older adults. As stated by Hofer et al. (19), internet use can be seen as a valuable resource for older adults to manage loss, especially for those facing more mobility or activity limitations. Network informatization has contributed significantly to improving the life satisfaction of people with mobility disabilities (20), and may help increase internet use and use of virtual media as a means of meeting people (21). Related research has shown that analysis of online media can be an effective way to understand the health experiences of people with functional disability (22). Therefore, under the multiple backgrounds of digitalization and aging, it is still necessary to pay attention to how the middle-aged and older groups benefit from the internet.

Internet penetration will have a certain degree of impact on mental health due to the effects of illness and functional disability in middle-aged and older adults. According to the internet compensation theory, internet use can help reduce DS by meeting people’s unmet psychosocial needs in real life (23). Studies have reported a significant association between internet use and DS in middle-aged and older adults (24). However, current research findings are inconsistent. Most studies confirm that internet use as a simple, convenient, and low-cost means of social engagement can reduce DS in middle-aged and older adults (25, 26). But some studies have also found no association between internet use and DS (27). It may even lead to more serious health outcomes, such as increased depression (28), increased social isolation (29), etc. The emergence of the digital divide among the information-disadvantaged groups has further expanded social inequality and alienation from daily social life (30). Part of it can be explained as the negative psychological health consequences caused by the accompanying social exclusion, social isolation, and lack of social participation (31). Thus, there remains insufficient evidence to assess the relationship between internet use and DS in middle-aged and older adults, particularly among those with functional disability.

Internet use has been shown to have a significant impact on DS in middle-aged and older adults, yet few studies have explored the potential mediating mechanisms by which internet use affects DS. In studying the relationship between internet use and DS, some scholars have studied how internet use affects DS. They found that internet use improved DS by enhancing factors including social capital, such as social engagement, social support, and interpersonal relationships (32–34). The existing literature suggests that a range of social factors are associated with internet use and DS in middle-aged and older adults. It is worth noting that social isolation has always been considered a risk factor for poor physical and mental health (35, 36). Social isolation is more common among middle-aged and older adults due to factors such as living alone, chronic illness, and functional disability (37, 38). Numerous studies have shown that social isolation puts middle-aged and older adults at great risk of depression (36, 39). Internet use plays an important role in alleviating social isolation (40). The internet can reduce social isolation by expanding social networks or increasing the frequency of interactions with existing acquaintances (41). A study showed that social networks of sufficient size, quality, and frequency of interaction benefit middle-aged and older adults physically and mentally (42). Its role may be particularly evident when persons are unable to integrate into social groups due to mobility issues (43). Furthermore, according to the information processing model, the internet has long been considered a cultural tool that influences cognitive processes and an environmental stimulus that contributes to the formation of specific cognitive architectures (44). For socially isolated middle-aged and older adults, internet use may mitigate the negative effects of social isolation on a cognitive level. A recent study found that among socially isolated older adults, higher internet use was associated with lower levels of depression (45). This situation can be explained as the previous internet use habits of older adults may buffer the impact of social isolation on increased levels of depression. It can be seen that social isolation is a possible mediator between internet use and DS. Although previous studies have shown an association between the internet, social isolation, and DS with each other (29, 36), few studies have discussed the potential role of social isolation in assessing the association between internet use and DS. It is unclear whether social isolation mediates the relationship between internet use and DS in middle-aged and older adults with functional disability.

Research remains divided on whether internet use has an impact on the health of middle-aged and older adults with functional disability. The purpose of the study is to explore the role of social isolation in the relationship between internet use and DS in Chinese middle-aged and older adults with functional disability. At the same time, the study will explore how they interact to reduce the risk of DS. The following research hypotheses are proposed: (1) Internet use is significantly associated with DS in middle-aged and older adults; (2) Social isolation mediates, to some extent, the relationship between internet use and DS in middle-aged and older adults with functional disability.

## 2. Methods

### 2.1. Data source and sample

This study is based on the data from Wave 4 of the 2018 China Health and Retirement Longitudinal Study (CHARLS). The nationally representative longitudinal survey began in 2011 and followed the sample in 2013, 2015, and 2018. The purpose of the survey is to investigate the health and economic adjustment of China’s rapidly aging population, including demographic characteristics, socioeconomic status, family relationships, health status, and health care. A multi-stage probability sampling method was used to select the sample, with the final sample drawn from 150 counties in 28 provinces in China. The further detailed description of the CHARLS data can be found in the cohort profile (46).

The 2018 CHARLS data selected for the study is the most recent wave of data available, covering 19,816 respondents aged 45 years and older in 450 communities (villages) in China. Considering the needs and objectives of the study, a total of 7,955 participants, including 3,845 males and 4,110 females, were included after excluding respondents under 45 years of age (age < 45 or missing date: 261) and participants with missing key variables (internet use missing data: 22, social isolation missing data: 9460, DS missing data: 2118). All interviewees were required to sign informed consent. Ethics approval for the data collection in CHARLS was obtained from the Biomedical Ethics Review Committee of Peking University (IRB00001052-11015).

### 2.2. Variables

#### 2.2.1. Independent variable

Internet use was selected as the independent variable. In the CHARLS 2018 questionnaire, the following question was used to measure internet use, where respondents were asked to answer the question “In the past month, have you used the internet?” By answering “yes” or “no.” We treated their responses as a dichotomous variable, recorded as “yes = 1” and “no = 0” (47). The types of functions used on the internet were also surveyed, with options including chat, watch news, watch videos, play games, financial management, and others. This study classifies chat as social needs, where middle-aged and older adults use the internet to keep in touch with their children, relatives, and friends. Watch news is information reception, where middle-aged and older adults use the internet to actively obtain information and integrate into society. Watch videos and play games are entertainment activities, which can enrich spiritual life. Financial management and others are the functions of daily life.

#### 2.2.2. Dependent variable

Researchers used the Center for Epidemiologic Studies Depression Scale (CES-D-10) to assess the severity of individual DS. A total of 10 items on the CES-D-10 assessed participants’ feelings during the previous week, such as worry, hope, fear, loneliness, unhappiness, attention deficits, and sleep disturbances. Respondents were asked to rate the frequency of each item by choosing one of four response options ranging from “0 = little or no time” to “3 = most or all of the time.” The sum of the scores ranges from 0 to 30, with higher scores indicating greater severity of DS. In this study, respondents with CES-D-10 ≥ 10 were categorized as having DS, whereas respondents with CES-D-10 < 10 were categorized as not having DS (48).

#### 2.2.3. Mediating variable

The four items were combined to form a score for social isolation (49). A score of 1 was assigned for each of the following: (1) Unmarried (never married, separated, divorced, widowed); (2) Having less than weekly contact with children (face-to-face, telephone, or email); (3) Living in a rural area, and (4) Not participating in any social activities in the past month (e.g., interacting with friends; playing chess or cards; participating in sports, social, or other clubs). Scores ranged from 0 to 4, with higher values indicating greater isolation. We categorized participants according to whether their scores were low (≤1) or high (≥2).

#### 2.2.4. Control variables

Based on previous literature, we controlled for possible covariates associated with DS in CHARLS. The respondents’ demographic information including gender, age (45–59, 60–69, and ≥ 70 years), education level (illiterate, elementary and middle school, high school and above), self-reported health status (good, not good), life satisfaction (very, fair, and poor), chronic disease status (no and yes), activities of daily living (as measured by the Katz Index of Independence in Activities of Daily Living), instrumental activities of daily living (as measured by Lawton’s Instrumental Activities of Daily Living), smoking (current smoked, ever smoked, and never smoked), and drinking status (current drink, ever drink, and never drink).

### 2.3. Statistical analysis

SPSS 26.0 and SPSS PROCESS (50) were used for statistical analysis. First, chi-square tests were used to describe differences in the prevalence of DS across different levels within the entire sample. Then, the study used multiple linear regression to explore the relationship between internet use and DS in middle-aged and older adults with functional disability. Also, to test the social isolation mediating effect, a pass-through analysis was conducted in PROCESS in SPSS, and the significance of the social isolation mediating effect was assessed using the Sobel test and corrected for bias by bootstrapping (bootstrap sample size of 5,000). The mediating effect was considered significant only when both the total and indirect effects were significant. Results are expressed as regression coefficients and their bootstrapped 95% confidence intervals (CI), with a significance level set at *p* < 0.05. The mediation analysis also controlled for all covariates.

## 3. Results

### 3.1. Participants characteristics

As shown in Table 1, a total of 7,955 participants were included in this study, of whom 1,001 (12.58%) used the internet, 3,379 (42.48%) had DS, and 4,808 (60.44%) were in high social isolation. When comparing characteristics with and without DS, the prevalence of depression was associated with age, gender, education level, chronic disease status, social isolation status, and internet use. The incidence of DS in middle-aged groups is higher than that in other age groups. Male and literate middle-aged and older adults are less likely to have DS than women and illiterate participants. The prevalence of DS was higher among participants with chronic medical conditions. Middle-aged and older participants who did not use the internet and were highly socially isolated had higher levels of DS.

**Table 1:** Characteristics of the sample by the percentage of depressive symptoms.

		Depressive symptoms		
Variable, *N* (%)	Total (*N* = 7,955)	Yes (*N* = 3,379)	No (*N* = 4,576)	*p*-value
**Demographic variables**				
Age				0.325
45–59	3,179 (39.96)	1,318 (39.01)	1861 (40.67)	
60–69	2,671 (33.58)	1,151 (34.06)	1,520 (33.22)	
≥70	2,105 (26.46)	910 (26.93)	1,195 (26.11)	
Gender				<0.001
Male	3,845 (48.33)	1,278 (37.82)	2,567 (56.10)	
Female	4,110 (51.67)	2,101 (62.18)	2009 (43.90)	
Education level				<0.001
Illiterate	3,472 (43.65)	1807 (53.48)	1,665 (36.39)	
Elementary & Middle School	3,524 (44.30)	1,337 (39.57)	2,187 (47.79)	
High School and above	959 (12.05)	235 (6.95)	724 (15.82)	
Self-rated health				<0.001
Good	5,615 (70.58)	1,838 (54.39)	3,777 (82.54)	
Not good	2,340 (29.42)	1,541 (45.61)	799 (17.46)	
Life satisfaction				<0.001
Very	2,630 (33.06)	718 (21.25)	1,912 (41.78)	
Fair	4,273 (53.71)	1,802 (53.33)	2,471 (54.00)	
Poor	1,052 (13.23)	859 (25.42)	193 (4.22)	
Smoking status				<0.001
Current smoked	2,183 (27.44)	813 (24.06)	1,370 (29.94)	
Ever smoked	1,232 (15.49)	423 (12.52)	809 (17.68)	
Never smoked	4,540 (57.07)	2,143 (63.42)	2,397 (52.38)	
Drinking status				<0.001
Current drink	2,654 (33.36)	926 (27.41)	1728 (37.76)	
Ever drink	312 (3.92)	137 (4.05)	175 (3.83)	
Never drink	4,989 (62.72)	2,316 (68.54)	2,673 (58.41)	
Chronic conditions				<0.001
No	1,449 (18.22)	391 (11.57)	1,058 (23.12)	
Yes	6,506 (81.78)	2,988 (88.43)	3,518 (78.88)	
ADL disability				<0.001
No	7,290 (91.64)	2,983 (88.28)	4,307 (94.12)	
Yes	665 (8.36)	396 (11.72)	269 (5.88)	
IADL disability				<0.001
No	6,749 (84.84)	2,578 (76.29)	4,171 (91.15)	
Yes	1,206 (15.16)	801 (23.71)	405 (8.85)	
Social isolation status				<0.001
Low isolation	3,147 (39.56)	1,096 (32.44)	2051 (44.82)	
High isolation	4,808 (60.44)	2,283 (67.56)	2,525 (55.18)	
Internet use				<0.001
Yes	1,001 (12.58)	278 (8.23)	723 (15.80)	
No	6,954 (87.42)	3,101 (91.77)	3,853 (84.20)	
**Types of internet usage functions**				
Social needs	677 (8.5)	192 (5.68)	485 (10.60)	<0.001
Information reception	781 (9.8)	211 (6.24)	570 (12.46)	<0.001
Recreational activities	710 (8.9)	205 (6.07)	505 (11.04)	<0.001
Daily life	207 (2.6)	48 (1.42)	159 (3.47)	<0.001

ADL, activities of daily living; IADL, instrumental activities of daily living.

### 3.2. Correlations

The results of the correlation analysis are shown in Table 2. Internet use was negatively associated with DS and social isolation. DS were positively correlated with social isolation. The significant correlations between the study variables provided a good basis for the subsequent hypothesis and mediation tests.

**Table 2:** Bivariate correlations between variables of interest.

Variables	1	2	3
1 Internet use	1		
2 Social isolation	−0.363***	1	
3 Depressive symptoms	−0.128***	0.166***	1

***p < 0.001.

### 3.3. The relationship between internet use and depressive symptoms

Table 3 shows the results of the multiple linear regression. As shown in Table 3, specific regression results are demonstrated by gradually putting in individual characteristic variables and social characteristic variables.

**Table 3:** Regression analysis of internet use and depressive symptoms in middle-aged and older adults.

Variable	Model 1	Model 2	Model 3	Model 4
Internet use	−2.559*** (0.225)	−0.993*** (0.206)	−0.931*** (0.205)	−0.493** (0.210)
**Age (ref: ≥70)**				
45–59		0.073 (0.165)	0.216 (0.168)	0.666*** (0.169)
60–69		0.088 (0.163)	0.093 (0.163)	0.374** (0.162)
**Gender (ref: male)**				
Female		1.731*** (0.132)	1.856*** (0.195)	1.834*** (0.193)
**Educational attainment (ref: high school and above)**				
Illiterate		2.483*** (0.224)	2.458*** (0.223)	2.075*** (0.224)
Elementary and middle school		1.134*** (0.210)	1.135*** (0.209)	0.984*** (0.208)
**Self-rated health (ref: good)**				
Not good		3.614*** (0.143)	3.401*** (0.146)	2.941*** (0.149)
**Life satisfaction (ref: fair)**				
Very		−2.461*** (0.140)	−2.425*** (0.139)	−2.431*** (0.138)
Poor		5.672*** (0.197)	5.627*** (0.196)	5.391*** (0.195)
**Smoking status (ref: never smoked)**				
Current smoked			0.555*** (0.198)	0.531*** (0.196)
Ever smoked			−0.127 (0.225)	−0.103 (0.223)
**Drinking status (ref: never drink)**				
Current drink			−0.221 (0.152)	−0.156 (0.150)
Ever drink			0.067 (0.331)	−0.034 (0.327)
**Chronic conditions (ref: no)**				
Yes			1.295*** (0.168)	1.292*** (0.167)
**ADL disability (ref: no)**				
Yes				0.666*** (0.236)
**IADL disability (ref: no)**				
Yes				1.792*** (0.194)
**Social isolation status (ref: low isolation)**				
High isolation				1.011*** (0.140)
*F*-value	128.929***	403.636***	266.980***	235.183***
*R* ^2^	0.016	0.314	0.320	0.335

**p < 0.05, ***p < 0.01. Values in brackets are standard errors.

The regression results of Model 1 showed that internet use was significantly and negatively associated with DS in middle-aged and older adults. After adding the personal characteristics and social characteristics variables sequentially, the regression coefficients of internet use are −0.993(*p* < 0.01), −0.931(*p* < 0.01), and − 0.493(*p* < 0.05) shown in Model 2, Model 3, and Model 4, respectively, still showed a significant association with DS in middle-aged and older adults. The results of this study support the conclusion that internet use can reduce DS in middle-aged and older adults. In addition, Model 4 shows the results of the effects of other control variables on DS in middle-aged and older adults. The effect of internet use on DS in middle-aged and older adults remained significant (*p* < 0.05). Regression results showed that gender, age, education level, self-rated health, life satisfaction, chronic disease status, ADL/IADL status, and social isolation were all associated with DS in middle-aged and older adults. Among them, females had higher levels of DS than males. The lower the level of education, the more severe the DS. The worse the self-rated health status and life satisfaction, the more pronounced the DS in middle-aged and older adults. The more severe the DS manifested in middle-aged and older adults with ADL/IADL disorders and high social isolation.

### 3.4. The relationship between the type of functioning based on internet use and depressive symptoms

As shown in Table 4, after controlling for age, gender, education level, and self-rated health confounding variables, social needs (B = −0.770, *p* = 0.041), and information reception (B = −1.067, *p* = 0.007) were negatively associated with DS in middle-aged and older adults. However, only Information reception (B = −3.161, *p* = 0.031) was significantly negatively associated with DS in middle-aged and older adults with functional disability.

**Table 4:** Regression results of the type of function based on internet use and depressive symptoms.

Variables	B	SE	*t*	*p*-value
**All participants (*N* = 7,955)**				
Social needs	−0.770	0.376	−2.047	0.041
Information reception	−1.067	0.394	−2.710	0.007
Recreational activities	0.259	0.405	0.641	0.522
Daily life	−0.854	0.461	−1.853	0.064
Constant term	6.577	0.207	31.744	<0.001
*F*-value	202.577			<0.001
*R* ^2^	0.169			
**ADL/IADL disabilities (*N* = 1,513)**				
Social needs	2.046	1.516	1.350	0.177
Information reception	−3.161	1.468	−2.153	0.031
Recreational activities	−1.022	1.583	−0.645	0.519
Daily life	0.152	1.564	0.097	0.922
Constant term	7.711	0.572	13.491	<0.001
*F*-value	42.143			<0.001
*R* ^2^	0.183			

SE, standard errors.

### 3.5. Analysis of mediating effects

**Figure 1:**
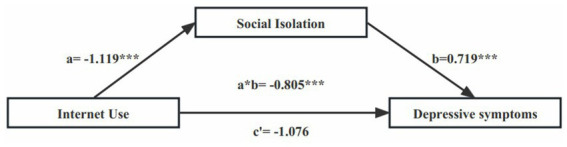
Statistical analysis of intermediary models (ADL/IADL disabilities) (***p < 0.001).

**Figure 2:**
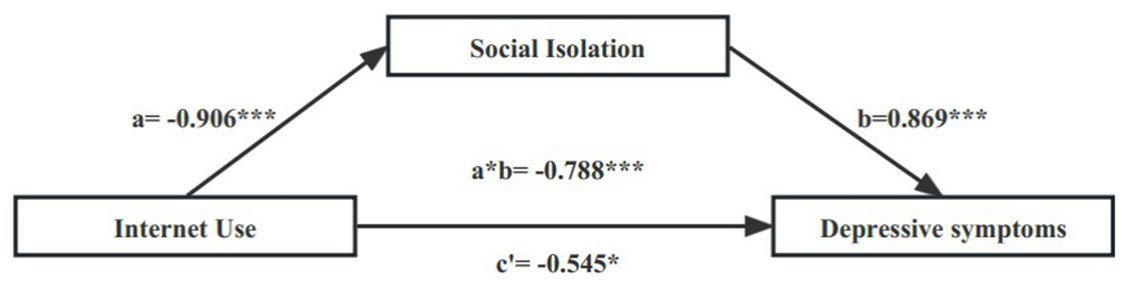
Statistical analysis of intermediary models (without ADL/IADL disabilities) (*p < 0.1, ***p < 0.001).

Mediating analysis was performed according to the presence or absence of ADL/IADL disabilities. The mediating effect of social isolation on internet use and DS in middle-aged and older adults is shown in Figures 1, 2. Adjusted for the following covariates: age, gender, al attainment, self-rated health, life satisfaction, smoking status, drinking status, and chronic conditions. As shown in Figure 1, social isolation fully mediates the relationship between internet use and DS in middle-aged and older adults with functional disability. As shown in Figure 2, social isolation partially mediates the relationship between internet use and DS in middle-aged and older adults with nonfunctional disability.

Table 5 lists the direct and indirect effects of internet use on DS in middle-aged and older adults under different ADL/IADL status, and the corresponding 95% confidence interval (CI). In conducting the mediation analysis, the following covariates were adjusted for age, gender, education level, self-rated health, life satisfaction, smoking status, alcohol consumption status, and chronic disease. Except for ADL/IADL disabilities, the direct effect was not significant, the other CIs did not contain 0, which indicated that the paths in the mediation model were basically significant. The direct effect of internet use on depression among middle-aged and older participants with functional disability was attenuated by social isolation (i.e., indirect effect = −0.805, *p* < 0.001, total effect = −1.881, *p* = 0.016). Among middle-aged and older participants without functional disability, social isolation partially mediated the effect between internet use and DS (direct effect = −0.545, *p* = 0.017, indirect effect = −0.788, *p* < 0.001, total effect = −1.333, *p* < 0.001).

**Table 5:** Mediating effects of social isolation between internet use and depressive symptoms in middle-aged and older adults with functional disabilities.

Model pathways	95% CI					
	Effect	Lower	Upper	SE	*t*	*p*-value
**ADL/IADL disabilities (*N* = 1,513)**						
Indirect effect	−0.805	−1.235	−0.406	0.221	−3.641	<0.001
Direct effect	−1.076	−2.654	0.502	0.805	−1.338	0.181
Total effect	−1.881	−3.414	−0.349	0.782	−2.407	0.016
**Without ADL/IADL disability (*N* = 6,442)**						
Indirect effect	−0.788	−0.945	−0.641	0.079	−9.984	<0.001
Direct effect	−0.545	−0.994	−0.096	0.229	−2.381	0.017
Total effect	−1.333	−1.762	−0.904	0.219	−6.088	<0.001

CI, confidence interval; SE, standard error.

## 4. Discussion

This study examined the impact of internet use on psychosocial health outcomes among middle-aged and older adults in the context of digital convergence and global aging. The findings suggest that internet use is associated with DS. Providing interventions to improve social isolation may help directly or indirectly reduce the degree of risk for DS in middle-aged and older adults with functional disability.

The study results showed that internet use had a significant effect on reducing the risk of DS in middle-aged and older adults, which is consistent with previous studies (51). This study validates the internet compensation theory that internet use plays an important role in improving mental health outcomes. Middle-aged and older adults, especially those with functional disability, face barriers to accessing health services and participating fully in society (52). For this group, the internet, as an emerging technology, can provide health care, entertainment, shopping information, etc., and enhance social connections. This may prompt middle-aged and older adults to adjust their psychological and social health barriers (53), thereby enhancing their sense of empowerment and independence (54), enabling them to have a better life experience and a higher level of health. Although the internet has the effect of improving DS. However, this study found that the number of middle-aged and older internet users is relatively limited. Middle-aged and older adults have the need to actively touch the internet, but there are still various obstacles in this process, such as the complexity of Apps, the inability to seek help, etc., (55). There is a lack of age-appropriate apps with simple interfaces and clear instructions to meet the demands of middle-aged and older adults. All these are not conducive to giving full play to the positive role of the internet. The relevant health departments should pay attention to cultivating the ability of this population to use the internet and increasing the internet penetration rate. Inevitably, internet use has negative effects. Considering the potential barriers to smartphone use (56) or the difficulty of indulging in the media environment due to the simple purpose of use and the short time of use (22), the impact of the internet on the mental health of middle-aged and older adults is moderate and positive.

Multifunctional internet use can potentially improve the DS of middle-aged and older adults. The study found that the social needs and information reception functions of internet use significantly reduced DS in middle-aged and older adults, with information reception being the most significant among functional disability groups. Research has shown that social media alone may not improve mental health in older adults (51). Touchscreen-based multifunction apps, including entertainment, transportation, social media-related, etc., may reduce the risk of DS in older adults (57). In contrast to the findings of Fan et al. (58) who hold that watch news had the least effect on depression levels in middle-aged and older adults. However, we discovered that information reception had the most significant effect on depression levels in middle-aged and older adults. Internet information acquisition can promote middle-aged and older adults to timely understand external information and the latest social development trends, so the influence degree of entertainment and social interaction gradually decreases. For people with limited mobility, this is especially beneficial (59). The abundance of information and resources on the internet makes it more conducive than traditional media (60) to improve the social isolation of middle-aged and older adults with functional disability (61). Middle-aged and older adults with functional disability can obtain more news about medicine, technology, and health through the internet, which will increase their frequency of contact with the outside world and reconnect with the environment. This may compensate to some extent for the social isolation in reality and thus improve their DS.

Social isolation fully mediated the relationship between internet use and DS in Chinese middle-aged and older adults with functional disability and partially mediated the relationship in middle-aged and older adults without functional disability. This implies that the internet reduces the risk of DS by improving social isolation in middle-aged and older adults with functional disability, which is similar to previous research findings (62). Middle-aged and older adults with limited mobility are more likely to experience loneliness and social isolation (63), and their likelihood of mental health risk is greater. The internet, as a coping mechanism for health challenges in later life, can be particularly helpful in keeping middle-aged and older adults who have limited mobility due to health conditions socially connected. It can play an important role in alleviating mental illnesses such as depression by building strong social networks for distraction from social isolation and painful emotions and directly enhancing their sense of self-worth (64). Given the cultural traditions that emphasize the importance of family and social networks, the link between social ties and health may be more pronounced in the Chinese population (65). Our findings also suggest that Chinese middle-aged and older adults with higher levels of social isolation, such as functional disability, may benefit more from internet use and that targeted internet health education is a possible safeguard to prevent or reduce the risk of DS. Future research on the relationship between different categories of functional disability and internet use among middle-aged and older adults needs to be more nuanced and detailed.

There are certain limitations of the study. First, the use of cross-sectional studies cannot monitor changes in mental health dynamics in middle-aged and older adults, and further longitudinal studies are needed to confirm this in the future to deepen the understanding of mental health. Second, DS in middle-aged and older adults are influenced by numerous factors. Further exploration of the different components of internet use on the health of middle-aged and older adults is needed to more clearly elucidate the potential mechanisms of internet use on mental health. Furthermore, participants’ DS were based on self-report measures, which may be influenced by personal feelings at the time, and thus bias may affect the results.

## 5. Conclusion

Under the background of accelerating global aging and expanding the scale of the older population, speeding up the digital integration of older adults, and narrowing the digital divide for older adults are the new demands and practical contents of promoting active aging in the digital society. Our findings determined that the impact of internet use on the mental health status of middle-aged and older adults can be predicted by social isolation status. For middle-aged and older adults with functional disability, internet use is critical for enhancing social relationships and maintaining their mental health status. The findings of this study highlight the need to develop and implement strategies or policies that promote or popularize internet use to mitigate mental health disparities among older adults in an internet society. Further research into the experiences of people with functional disability could lead to a broader survey of health care and policy interventions to address the health needs that may exist in the current global health crisis.

## Data availability statement

The datasets presented in this study can be found in online repositories. The names of the repository/repositories and accession number(s) can be found at: http://charls.pku.edu.cn/pages/data/2018-charls-wave4/zh-cn.html.

## Author contributions

MW, CL, XZ, TH, LZ, and YY conceived and designed the study, performed all statistical analyses and interpreted the data, drafted and revised all parts of the manuscript. MW drafted and revised the manuscript. MW, FY, and YH revised the discussion part and data processing of result part (including data regression analysis, mediation effect analysis). YH revised and provided financial support for the progress of the manuscript. All authors contributed to the article and approved the submitted version.

## Funding

This research was funded by Hubei Provincial Department of Education Philosophy and Social Science Foundation (22Y089).

## Conflict of interest

The authors declare that the research was conducted in the absence of any commercial or financial relationships that could be construed as a potential conflict of interest.

## Publisher’s note

All claims expressed in this article are solely those of the authors and do not necessarily represent those of their affiliated organizations, or those of the publisher, the editors and the reviewers. Any product that may be evaluated in this article, or claim that may be made by its manufacturer, is not guaranteed or endorsed by the publisher.

## Abbreviations

DS: depressive symptoms
CHARLS: the China Health and Retirement Longitudinal Study
ADL: activities of daily living
IADL: instrumental activities of daily living
CI: confidence interval
